# Aortic-Valve Sparing Repair and Homograft and Dacron Pulmonary Reconstruction Long-term After Arterial Switch Operation

**DOI:** 10.1016/j.cjcpc.2022.06.001

**Published:** 2022-06-17

**Authors:** Mohsyn Imran Malik, Ahmed Hafiz, Ali Hage, Kambiz Norozi, Michael W.A. Chu, Lin-Rui Guo

**Affiliations:** a Division of Cardiac Surgery, Schulich School of Medicine and Dentistry, London, Ontario, Canada; b Division of Pediatric Cardiology, The Children’s Hospital, London, Ontario, Canada

## Abstract

The arterial switch operation is the gold-standard treatment for dextro-transposition of the great arteries. Long-term follow-up data are beginning to reveal its natural history and associated late complications, including various reoperations for those complications. Given the unique anatomy and the increasing longevity of these patients, there is a need for effective surgical repair options to address aneurysmal and degenerative changes in both neoaortic and pulmonic roots. Thereby, we describe our technique and the novel considerations for prosthetic choice with reconstruction of both the neoaortic root and pulmonary artery, with satisfactory postoperative results.

## Résumé

La détransposition artérielle constitue le traitement de référence dans les cas de dextro-transposition des gros vaisseaux. De nouvelles données, issues du suivi à long terme, nous permettent de mieux comprendre l’évolution naturelle à la suite de cette intervention et les complications tardives qui y sont associées, y compris les diverses interventions à réaliser pour les corriger. Étant donné les caractéristiques anatomiques uniques de ces patients et l’augmentation de leur espérance de vie, il est nécessaire de proposer des options efficaces de réparations chirurgicales pour remédier aux changements anévrismaux et dégénératifs des racines néoaortique et pulmonaire. Ainsi, nous décrivons la technique que nous avons utilisée et les nouveaux éléments qui entrent en ligne de compte dans le choix d’une prothèse pour une reconstruction de la racine néoaortique et de l’artère pulmonaire, avec des résultats postopératoires jugés satisfaisants.

**Figure 1:**
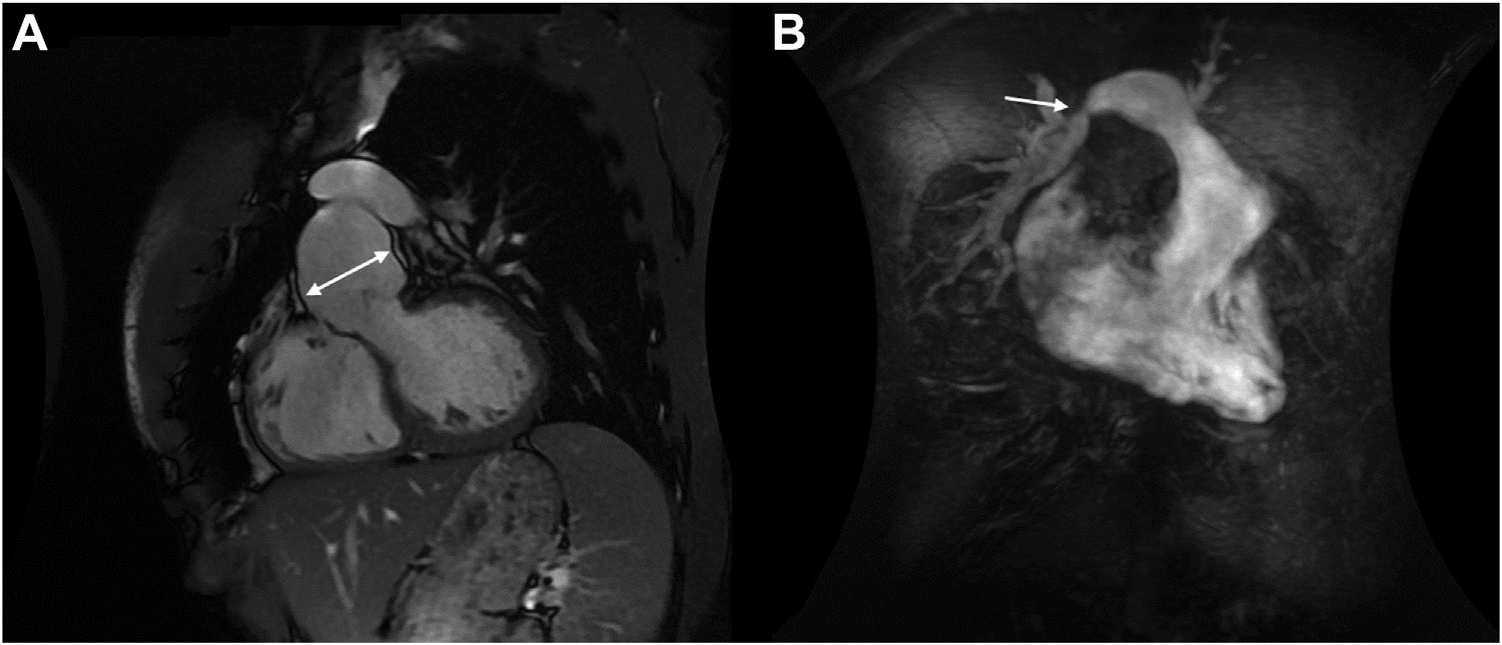
Cardiac magnetic resonance imaging. (**A**) Demonstration of the left-ventricular outflow tract with severe dilatation of the aortic root. (**B**) Severely enlarged right ventricle with the pulmonary artery and branches. The **arrow** identifies the area of right pulmonary artery stenosis.

An asymptomatic 28-year-old man with a history of dextro-transposition of the great arteries and arterial switch operation (ASO) after birth was found on routine follow-up with a congenital cardiologist to have progressive severe pulmonary regurgitation with dilated right ventricle (RV) and an aortic root aneurysm, measuring a maximum diameter of 55 mm on magnetic resonance imaging, with moderate aortic regurgitation (Fig. 1). There was a restrictive ventricular septal defect (VSD) and right PA (RPA) stenosis with a gradient of 30 mm Hg.

The patient was taken to the operating room for an elective redo-sternotomy. The chest was opened, and scar tissue was dissected. Sufficient mobilization was achieved to free the RV, right atrium, and ascending aorta. The RPA crossed the aorta anteriorly, an anatomic result of a LeCompte manoeuvre from the ASO. Cardiopulmonary bypass was initiated with cannulations to the right femoral artery and right atrium. Cardioplegia was delivered into the aortic root and subsequently via direct coronary ostial cannulations.

The aorta was transected above the sinotubular junction. A 3-mm VSD was identified at the membranous septum. The VSD was associated with a redundant fibrous rim, and thus, it was primarily closed with 2 interrupted 4-0 Prolene sutures. The root was aneurysmal, and the aortic annulus was measured 27 mm. The aortic valve was tricuspid with normal geometric heights. We therefore proceeded with a valve-sparing operation with the reimplantation technique. Both coronary buttons were dissected, and the aortic root was circumferentially skeletonized down to the ventricular-aortic junction. Using a row of nine 2-0 braided subannular sutures, the aortic valve was reimplanted within a 30-mm Valsalva graft (Terumo, Inchinnan, United Kingdom), tying the annuloplasty sutures around a 24-mm Hegar dilator and a second layer of running 4-0 Prolene starting at each nadir and running up to the top of all 3 commissures. The coronary buttons were sewn to the neoaortic root with 5-0 Prolene running sutures.

The PA was transected at the bifurcation. The pulmonary cusps were tricuspid, but thickened, shortened, and not amenable to repair. The native main PA and valve were resected. A 30-mm pulmonary homograft (Cryolife, Kennesaw, GA) was tailored and anastomosed to the native pulmonary bifurcation. The proximal homograft was anastomosed to the outflow tract of RV with running 4-0 Prolene sutures.

The native ascending aorta was transected distally below the cross-clamp and anastomosed to the root graft with running 4-0 Prolene sutures.

**Figure 2:**
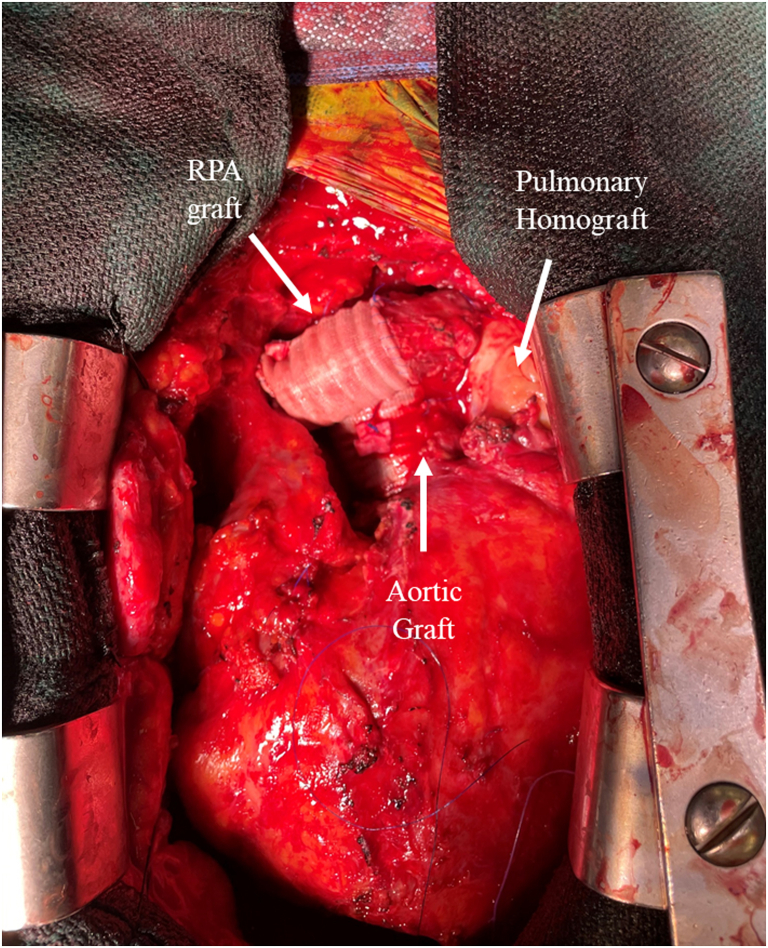
Completed reconstruction of the aortic root, the ascending aorta, the main pulmonary artery, and the right branch of the pulmonary artery. RPA, right pulmonary artery.

The RPA was distally stenotic, secondary to tenting from the ascending aorta. The RPA was transected, and a 4-cm-length 16-mm Dacron interposition graft was inserted with running 5-0 Prolene sutures, which completed the repair (Fig. 2). Cross-clamp time and cardiopulmonary-bypass time were 220 minutes and 271 minutes, respectively.

The patient recovered well without complications and was discharged home on the fourth postoperative day. His transthoracic echocardiograms at discharge and at 3-month follow-up showed normal biventricular function, with no residual VSD. There was no aortic regurgitation with a peak gradient of 7 mm Hg. The pulmonary homograft was well seated with no regurgitation or stenosis.

## Discussion

Long-term follow-up reports for ASO have shown excellent survivals of 87% survival at greater than 20 years.^1^ Unfortunately, late complications also start to arise as the patients enter the third decade of life. These include right-sided lesions such as pulmonary valve regurgitation and branch PA stenosis and left-sided lesions such as neoaortic regurgitation and neoaortic root dilatation as presented in our case.^1^, ^2^

We elected to preserve his aortic valve with the reimplantation technique as one would do given the anatomy. We decided further to reduce the aortic annulus diameter from 27 mm to 24 mm by tying subannular sutures against the 24-mm Hegar dilator to increase leaflet coaptation heights to achieve an optimal result with a longevity for this young patient.^3^

We routinely use pulmonary homografts to replace nonrepairable pulmonary valves, rather than other bioprosthetic valves for young patients. Recent data in redo-tetralogy patients for pulmonary regurgitation have shown that homografts had significantly lower transvalvular gradients on follow-up and lower rates of reintervention for structural valve degeneration, compared with bioprosthetics.^4^

After taking off the cross-clamp, we accidentally found that the rather stiff Dacron aortic graft exacerbated compressive tenting of RPA leading to more RPA stenosis, a consequence of the posterior positioning of the aorta in relation to the RPA.^5^ We opted to lengthen the RPA with an interposition Dacron graft.

In ASO patients who require complex repairs of both great arteries as in this case, careful preoperative evaluations, understanding the unique anatomy, and explicit planning are the keys to the optimal results of this type of redo surgery. This case provides a rationale to approach multilesion ASO late complications though further reoperative data are needed for favourable long-term outcomes.

**Novel Teaching Point**

• For arterial switch operation patients requiring reoperation for lesions of the great arteries, there are important and novel considerations with regards to longevity, anatomy, and prosthetic choice to ensure continued satisfactory long-term results.
